# Effect of a Repeated Sprint Ability test on the muscle contractile properties in elite futsal players

**DOI:** 10.1038/s41598-018-35345-z

**Published:** 2018-11-23

**Authors:** Javier Sánchez-Sánchez, David Bishop, Jorge García-Unanue, Esther Ubago-Guisado, Enrique Hernando, Jorge López-Fernández, Enrique Colino, Leonor Gallardo

**Affiliations:** 1 School of Sport Sciences, Universidad Europea de Madrid, Villaviciosa de Odón, (Madrid), Spain; 2 Institute of Sport, Exercise and Active Living (ISEAL), Victoria University, Melbourne, Australia; 3 School of Medical & Health Science, Edith Cowan University, Joondalup, Australia; 4 IGOID Research Group, University of Castilla-La Mancha, Toledo, Spain

## Abstract

The aim of this study was to evaluate the effect of a repeated sprint ability (RSA) test on the contractile properties of the muscles in elite futsal players. A total of 20 elite players completed the RSA test (7 × 30 m), and the contractile response from the rectus femoris (RF) and biceps femoris (BF) of both legs were analysed pre and post through tensiomyography. There was a significant increment in 30-m times from the third sprint onwards (*p* < 0.05). The percent decrement in sprint ability (RSA_DEC_) with respect to the first sprint was significantly higher in the last sprint. The players did not show evidence of lateral asymmetry in any of the muscle groups analysed after the RSA test (*p* > 0.05). Following the RSA test there was a significant reduction in the delay time (Td) in RF, a significant decrement in half-relaxation time (Tr) in the RF, and a significant reduction in sustain time (Ts) in the RF and BF of both legs. The maximum radial displacement of the muscle belly (Dm) increased (1.6 mm; effect size = 0.75; *p* < 0.05) in the RF after the RSA test, indicating reduced muscle stiffness and the ability to generate strength rapidly. The decrement in performance during the RSA test was significantly correlated with changes in contraction time (Tc) in RF and BF, Td in BF, and Dm in RF (*p* < 0.05). The RSA test generated alterations in the contractile properties of the RF and BF in elite players. However, futsal players did not present asymmetries in any muscular parameters. The baseline contractile muscle parameters could be an important factor related to performance of players during repeated high-intensity actions.

## Introduction

Futsal is a sport of intermittent efforts, with aerobic and dynamic components, that requires a player to be at 85% of their maximum heart rate or above during most of the playing time^1–5^. Although the physical, physiological, technical, and tactical demands have not been fully studied, researchers agree that the ability to develop and maintain high-intensity efforts and sprint performance over time has a direct influence on the match performance^1,2,4–6^. Consequently, the use of repeated sprint ability test (RSA) is recommended to evaluate the ability of athletes to cope with the demands of competition^7,8^. It has been shown that the performance of the athlete in a RSA test offers relevant information on the explosive ability of footballers^8^.

The RSA test is recognized as a valid method to reproduce performance decrement and fatigue in soccer players^9^. Fatigue of the lower-limb muscles appears to be an important factor elevating the risk of injury^10,11^. This suggests the behaviour of the lower-limb muscles after fatigue-inducing actions may be an effective way to identify factors related to injury risk^12^. Some of these factors include muscle stiffness, contraction speed, or displacement of the muscle belly^13^. These mechanical and contractile variables of the muscle can be determined by Tensiomyography (TMG), via the application of an electrical stimulus^14^.

The TMG technique has been reported to have high reproducibility and reliability to measure values like contraction time (Tc), half-relaxation time (Tr), delay time (Td), sustain time (Ts), and maximum radial displacement of the muscle belly (Dm) for the medial vastus, lateral vastus, femoris rectus, and femoris biceps muscles^13,15–17^. Therefore, TMG has been identified as a reliable method for the identification of muscular lateral asymmetries between dominant and non-dominant limbs in the lower-limb muscles^18^, which is related to the risk of injury and the stretch-shortening cycle efficacy, especially in sports in which limb dominance plays a factor^19–21^. Also, TMG has been described as a reliable method for the identification of differences in muscle responses after completing fatiguing efforts like the RSA test^21^.

Thus, the aim of this study was to evaluate the effect of a RSA test on the contractile properties of the muscles in elite futsal players. It was hypothesized that lower-limb muscle activation times (Tc, Td, and Ts) would be correlated with better results in the RSA test. We also hypothesised that elite futsal players would not show muscle contractile asymmetries between the dominant and non-dominant leg^22,23^. The results of this research will improve understanding of the acute effects of a repeated sprint test on the muscular response of elite futsal players.

## Method

### Participants

The sample was composed of two teams from the Spanish National Futsal League (LNFS). The results for a total of 20 players (25.5 ± 6.1 years; 176.9 ± 5.2 cm; 74.9 ± 5.2 kg; 13.1 ± 2.4% body fat) were included in this study. Contact with the clubs was carried out through the LNFS, with whom an agreement was signed for the conduct of this study. The study protocol was approved by the Local Ethics Committee (Hospital of Toledo), and was conducted in accordance with the Code of Ethics of the World Medical Association (Declaration of Helsinki). All of the participants signed an informed consent form in which the test procedures and possible risks were explained. In this document, the players also indicated their dominant leg.

### Experimental design

During the competitive period in the break for national team competitions, every team arranged 3 days with the researchers within the given period to allow the players to perform the proposed tests. On the first day, players performed an initial pilot test to become familiar with the tests included in the study protocol. During the second day, players were not allowed to perform any exhaustive activity to guarantee 24 h of rest before testing. Finally, all tests were carried out during the third day. At the beginning of the testing session, before the warm-up, each player attached a heart rate monitor (Firstbeat Technologies Ltd., Finland).

### Experimental protocol

#### Body composition

Both fat mass (g and %) and lean mass (g) of both legs was stimated using bioelectrical impedance (Tanita BC418-MA, Tanita Corp., Tokyo, Japan). The SECA scale (model 711; SECA GmbH & Co, KG, Hamburg, Germany) was used to measure the height of the participants.

#### Tensiomyography (TMG)

**Figure 1:**
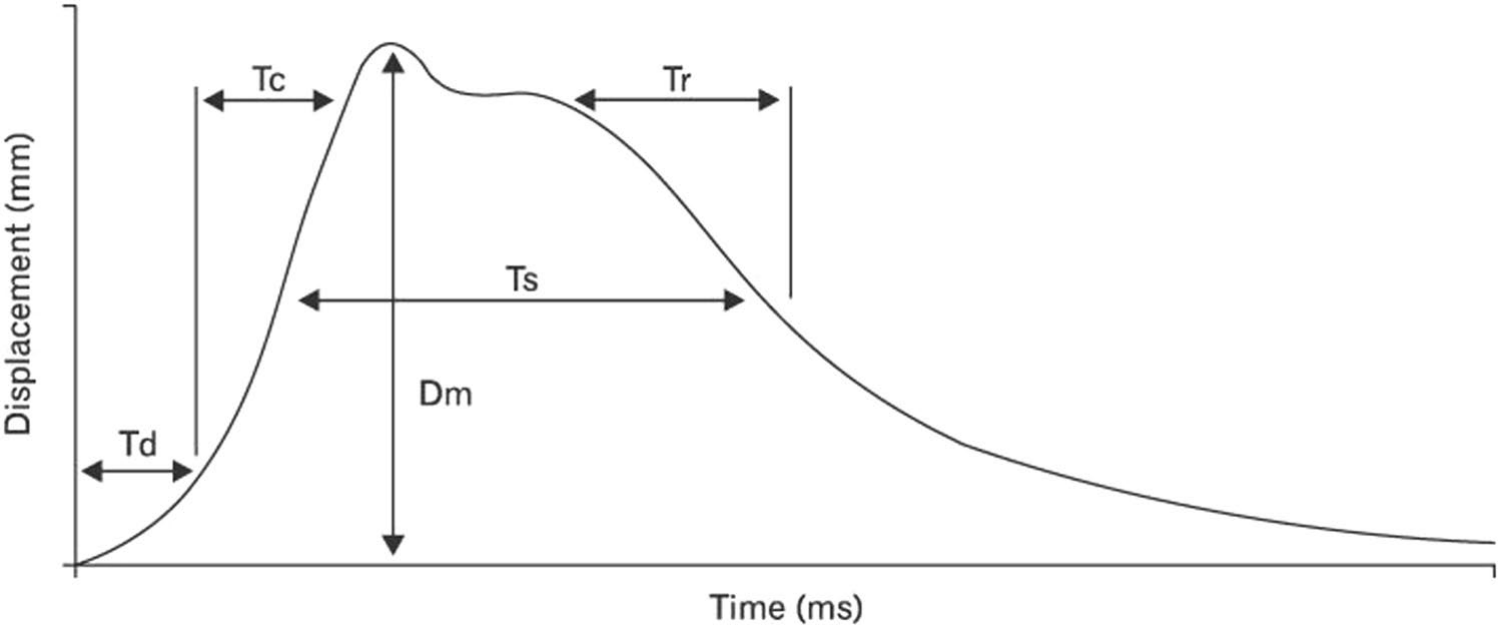
An example of how the TMG parameters were calculated (Carrasco *et al*.^15^). Td: delay time; Tc: contraction time; Ts: sustain time; Dm: maximum radial displacement of muscle belly; Tr: half-relaxation time.

Muscle response and lateral symmetry of both the rectus femoris (RF) and biceps femoris (BF) were assessed using tensiomyography (TMG-100 System electrostimulator, TMG-BMC d.o.o., Ljubljana, Slovenia). These muscles were selected because they are the most common muscles assessed in studies targeting soccer players due to their role in actions like jumping and kicking (BF) or in running and knee stabilisation (RF)^13^. This assessment provided the following information: the maximum radial displacement of the muscle belly (Dm), contraction time (Tc), delay time (Td), sustain time (Ts), and half-relaxation time (Tr) of these muscles under basal conditions (Fig. 1). These measurements were repeated in the same room immediately after (>1 min) the RSA test. The duration of the stimulus was 1 ms at several intensities (25, 50, 75 and 100 mA), following the protocol carried out in previous studies^18^. The properties of the rectus femoris were measured with the participant in a supine position, and with the knees flexed at 120 degrees with the help of a triangular foam cushion. The properties of the BF were measured with the participant in the prone position, and with the knee flexed at 5 degrees with the help of a foam cushion^16^.

The muscle response was measured by placing a digital Dc-Dc transducer Trans-Tek® (GK 40, Panoptik d.o.o., Ljubliana, Slovenia) perpendicular to the muscle belly, along with two self-adhesive electrodes (TMG electrodes, TMG-BMC d.o.o. Ljubljana, Slovenia) placed equidistant at a distance of 50 to 60 mm from the digital transducer, since the distance between electrodes can vary the results^24^, the positions of the sensor and the electrodes were marked with a permanent marker to ensure that all measurements were performed at the same point and between 50 to 60 mm from the digital transducer. All measurements were carried out by the same expert technician. Krizaj *et al*.^25^ reported a low error level (0.5 to 2.0%) and a high reproducibility (ICC: 0.85–0.98) for the five parameters measured in this study (ICC: Dm = 0.98; Tc = 0.97; Td = 0.94; Ts = 0.89; Tr = 0.86).

#### Repeated sprint ability (RSA) test

The RSA test included seven repeated sprints of 30 m, with 20 s of active recovery between each sprint. Three pairs of photocells (Witty, Microgate, Bolzano, Italy) placed at 0, 5 and 30 m were used to assess performance in this test. This test was performed according to the methodology proposed in previous studies^26^. The best sprint time (RSA_BEST_), the mean time (RSA_MEAN_), the total time (RSA_TT_), the percent sprint decrement (RSA_DEC_ = ((total sprint time − best time*7)/best time ∗ 7) ∗ 100), and the percent difference from best and worst sprint during the RSA test (RSA_CHANGE_ = ((worst time − best time)/best time) ∗ 100) were also calculated^27,28^. Before the RSA test, participants carried out a standardised warm-up consisting of 5 minutes of running, 5 minutes of joint mobility, and three 30-m sprints of increasing intensity. The warm-up concluded with two 30-m sprints at maximum intensity separated by 4 minutes of active recovery (participants had to walk during the resting time). These two previous sprints performed in the warm-up were used as a control measure to guarantee players performed the RSA test at maximum speed. If the time of the first sprint of the RSA test was higher (>5%) than the best individual sprint performed prior to the beginning of the test, the RSA test was not considered valid and the player had to repeat the test after 5 min of recovery.

### Statistical analyses

SPSS 21.0 was used for the data analysis. A descriptive analysis (mean ± SD) of the tensiomyography test results and the performance parameters of the RSA test was performed. The Kolmogórov–Smirnov test showed a normal distribution of the variables. Two-way analysis of variance (ANOVA) was used to analyse the difference in the tensiomyography variables as a function of the time (pre and post) and dominance (dominant leg and non-dominant leg). The exercise-induced change in the TMG variables (percentage of change of the post with respect to the baseline tensiomyographic values) was also calculated. In addition, the confidence interval and the effect size (ES; Cohen’s d) of the pre to post differences for all variables (CI of 95%) was calculated. The ES was evaluated with the following criteria: 0 to 0.2 = trivial, 0.2 to 0.5 = small, 0.5 to 0.8 = moderate, and >0.8 = large^29^. The RSA data were analysed by one-way repeated measures ANOVA. A Bonferroni post-hoc test was used to study pairwise differences. A linear correlation (Pearson’s r) was calculated between the results of the RSA test and the tensiomyography variables derived from the dominant leg of the futsal players. Correlations were evaluated with the following criteria: 0 to 0.1 = trivial, 0.1 to 0.3 = small, 0.3 to 0.5 = medium, 0.5 to 0.7 = large, 0.7 to 0.9 = very large and 0.9 to 1.0 = nearly perfect^30^. The level of significance was established at *p* < 0.05.

## Results

**Figure 2:**
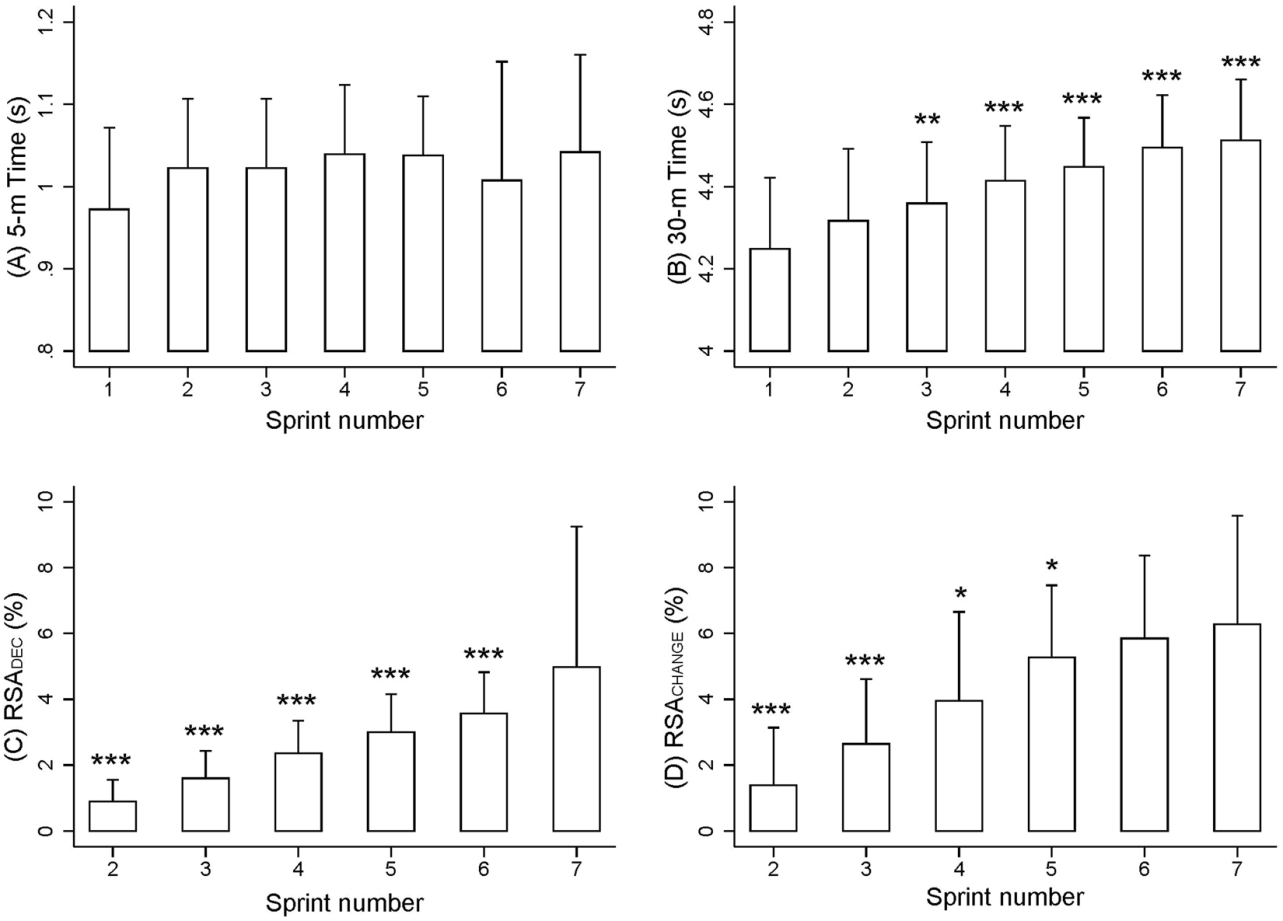
5-m (**A**) and 30-m (**B**) time and performance deterioration profile; RSA_DEC_ (**C**) and RSA_CHANGE_ (**D**) for the RSA test (7 × 30 m). RSA_CHANGE_: ((worst time − best time)/best time) * 100). RSA_DEC_: ((total sprint time − best time * 7)/best time * 7) * 100). *p < 0.05; *p* < 0.01; ***p < 0.0015; significantly different from the 1^st^ sprint for 30-m times; and significantly different from the 7^th^ sprint for RSA_DEC_ and RSA_CHANGE_ (n = 20). Data are presented as mean and SD.

The players had a RSA_TT_ of 29.9 ± 2.5 s, a RSA_MEAN_ of 4.4 ± 0.1 s, and a RSA_BEST_ of 4.2 ± 0.2 s. There were no significant differences between the times of the seven sprints during the first 5 m of each sprint (Fig. 2a; *p* > 0.05). However, compared with the first sprint, there was a significant increment in 30-m times from the third sprint onwards (*p* < 0.05; Fig. 2b). The percent decrement in sprint ability (RSA_DEC_) with respect to the first sprint was significantly higher in the last sprint (*p* < 0.05; Fig. 2c). There was not a significant decrease in performance in the seventh sprint (6.3 ± 3.3%) compared to the sixth sprint (5.9 ± 2.5%) for RSA_CHANGE_ (Fig. 2d).

In Table 1, the results of the tensiomyography for the RF and the BF (before and after the RSA test for both the dominant (D) and non-dominant (ND) leg of the futsal players) are presented. The results reveal an absence of significant differences between the dominant and non-dominant leg in the variables analysed (*p* > 0.05). However, following the RSA test, there was a significant reduction in the Td (D: 1.1 ms [CI: 0.02–2.3]; ES: 0.62; ND: 1.3 ms [CI: 0.2–2.4]; ES: 0.77), Ts (D: 53 ms [CI: 23.5–82.4]; ES: 1.22; ND: 45.4 ms [CI: 15.9–74.8]; ES: 0.96), Tr (D: 27.5 ms [CI: 3.6–51.5]; ES: 0.76; ND: 32.2 ms [CI: 8.3–56.1]; ES: 0.84) in the RF and the Ts (D: 58.9 ms [CI: 26.2–91.6]; ES: 1.90; ND: 53.5 ms [CI: 20.8–86.2]; ES: 0.83) in the BF in both the dominant and non-dominant legs. On the other hand, the Dm of the RF showed higher values after the RSA test in the dominant leg (1.6 mm [CI: 0.03–3.1]; ES: 0.75; *p* < 0.05).

**Table 1:** Results of the tensiomyography before (pre) and after (post) the RSA test for both the dominant and non-dominant leg.

	Dominant		Non-dominant	
	Pre	Post	Pre	Post
**RF**				
Td (ms)	23.9 ± 2.2*	22.8 ± 1.4	23.4 ± 1.8*	22.2 ± 1.6
Tc (ms)	31 ± 8.4	27.6 ± 3.8	28.3 ± 6.1	26 ± 4.8
Ts (ms)	125 ± 53.4*	72 ± 33.2	120.8 ± 60.4*	75.5 ± 33.8
Tr (ms)	61.6 ± 40.4*	34.1 ± 31.9	66.3 ± 47.6*	34.1 ± 29.3
Dm (mm)	6.9 ± 2.5*	8.4 ± 1.7	7.4 ± 3	7.7 ± 2.5
**BF**				
Td (ms)	23.2 ± 1.6	22.7 ± 1.6	22.4 ± 1.9	22.3 ± 1.7
Tc (ms)	26.3 ± 5.9	28.6 ± 11.6	30.6 ± 14.6	29.5 ± 13.3
Ts (ms)	205.5 ± 44.3*	146.6 ± 17.6	213.2 ± 73.1*	159.6 ± 56.3
Tr (ms)	60.4 ± 42.6	45.8 ± 27.7	65.1 ± 37.9	52.2 ± 46.2
Dm (mm)	5.8 ± 2.1	5.8 ± 2.1	5.6 ± 2.8	5.2 ± 2.5

*Significantly different from post (*p* < 0.05); Td = delay time, Tc = contraction time, Ts = sustain time; Tr = half-relaxation time; Dm = maximum radial displacement of muscle belly; RF = rectus femoris; BF = biceps femoris. n = 20 for all parameters.

The correlational analysis did not reveal any significant relation between the tensiomyography parameters in the RF prior to the RSA test and the results obtained in the test (*p* > 0.05). However, the Ts values obtained in the BF (Table 2) showed a positive relation with the best sprint time achieved during the RSA test (*r* = 0.476) and the mean sprint time (*r* = 0.528). There was a significant correlation between Td baseline values in BF and RSA_DEC_ (*r* = 0.487) and RSA_CHANGE_ (*r* = 0.591). Regarding the performance decrement, the RSA_DEC_ and the RSA_CHANGE_ were significantly correlated with the percent change of the Tc (*r* = −0.498) and Dm (*r* = −0.485), respectively, from pre to post the RSA test in the RF (Table 3). Finally, in the BF, the percent changes in the Td and Tc values from pre to post the RSA test were related with the deterioration in the sprint times during the test, independent of the formula used (Table 3; *p* < 0.05).

**Table 2:** Correlation coefficients for the baseline values of the tensiomyography in the BF and the total time (RSA_TT_), the best sprint time (RSA_BEST_), the mean time (RSA_MEAN_), the percent sprint decrement (RSA_DEC_), and the percent difference from best to worst sprint (RSA_CHANGE_) during the RSA test.

	RSA_TT_	RSA_BEST_	RSA_MEAN_	RSA_DEC_	RSA_CHANGE_
Td (BF_pre_)	0.032	−0.175	−0.007	0.487*	0.591**
Tc (BF_pre_)	0.254	−0.131	0.009	0.391	0.242
Ts (BF_pre_)	0.334	0.476*	0.528*	−0.202	−0.050
Tr (BF_pre_)	0.191	−0.111	−0.038	0.226	0.321
Dm (BF_pre_)	0.297	0.038	0.007	−0.105	−0.263

*p < 0.05; **p < 0.01; ***p < 0.001; Dm = maximum radial displacement of muscle belly; Tc = contraction time, Td = delay time, Ts = sustain time; Tr = half-relaxation time; BF = biceps femoris (n = 20).

**Table 3:** Correlation coefficients between the RSA_DEC_ and RSA_CHANGE_ derived from the RSA test and the percentage change in the TMG parameters from pre to post the RSA test for the RF and BF.

RF	RSA_DEC_	RSA_CHANGE_	BF	RSA_DEC_	RSA_CHANGE_
Td_CHANGE_	−0.197	−0.043	Td_CHANGE_	0.477*	0.578**
Tc_CHANGE_	−0.498*	−0.263	Tc_CHANGE_	0.497*	0.469*
Ts_CHANGE_	−0.186	−0.217	Ts_CHANGE_	−0.337	−0.318
Tr_CHANGE_	−0.158	−0.232	Tr_CHANGE_	−0.015	0.116
Dm_CHANGE_	−0.424	−0.485*	Dm_CHANGE_	0.184	0.018

*p < 0.05; **p < 0.01; ***p < 0.001; Dm = maximum radial displacement of muscle belly; Tc = contraction time, Td = delay time, Ts = sustain time; Tr = half-relaxation time; RF = rectus femoris; BF = biceps femoris. n = 20.

## Discussion

The results of this study revealed that the RSA test causes an acute alteration in the mechanical muscle parameters in the RF and the BF. This alteration is characterised by a change in the excitability of the RF in both legs (decrease of the Td, Tc, Ts and Tr), whilst its effect on the contractile properties of the BF was less clear. Correlations were found between the performance decrement of the RSA test and some of the tensiomyography parameters. In the RF, we observed negative correlations between RSA_DEC_ and Tc and between RSA_CHANGE_ and Dm. In the BF, negative correlations were found between both performance decrement indices (RSA_DEC_ and RSA_CHANGE_) and pre to post change of Td and Tc. No significant differences in TMG parameters were found between the dominant and non-dominant leg either before or after the RSA test. Therefore, it can be concluded that no lateral asymmetry exists regarding the contractile properties of the RF and the BF in the professional futsal players recruited for this study, neither at baseline nor after the RSA test.

Regarding RSA test performance, compared with the first sprint there was a significant increase in 30-m sprint time from the third sprint onwards. This is consistent with the results of previous studies on elite football players^27^ that have reported a significant increase in sprint time from the second sprint. The percent sprint decrement (4.98%) was similar to those reported in other repeated-sprint ability tests conducted with elite football^31,32^ and female futsal^33^ players. Oliveira *et al*.^4^ observed a higher sprint decrement (6.7%) in high-level futsal players, probably due to the inclusion of changes of direction in the protocol, a greater sprint distance (40 m), and the moment of the season (pre-season). In this study, the RSA_CHANGE_ was significant in the first five sprint, but no significant differences were observed among sprints 6 and 7. The effect size of the differences was lower from the fourth sprint, coinciding with Da Silva *et al*.^31^ who reported no significant differences between the last four sprints.

The baseline values of the TMG variables in elite futsal players differ from the values previously reported for recreationally-active populations^15,34^, ultra-endurance athletes^12^, and even professional football players^13,23^. These outcomes show that the TMG profile of futsal players is different to that of other sports and recreationally active populations. Futsal players showed lower Tc and Td in the RF muscle than these groups, suggesting a better contractile ability. This means that the contractile properties of futsal players are phenotypically faster than those found in other sports^35^. Tc, or contraction time, is defined as the time interval between the onset of development of twitch force and its peak. This parameter reflects the speed of force generation and is related with the slow and fast motor units^36^. Concretely, Tc is positive correlated with the proportion of type I fibres^37^. Therefore, athletes from sports that need better contractile performance show lower Tc values^35^. In the BF, the contractile properties were similar to elite football players^13^.

The correlation analysis between the baseline values of the TMG for the BF and RSA_TT,_ RSA_BEST,_ and RSA_MEAN_ revealed a medium correlation (0.3–0.5) between the Ts and the best sprint and a large correlation (0.5–0.7) between the Ts and the average sprint time. This suggests a better RSA (lower sprint time) in those players with lower Ts values. However, caution is required when interpreting this result due to the possible influence of the co-activation of other neighbouring muscles during the TMG evaluation^38^ and the low reliability of Ts^39^. There was also a significant positive correlation between delay time of the BF and RSA_DEC_ and RSA_CHANGE_. Td, or delay time, is the time lapse from when the impulse is transmitted to the muscle until when the displacement of the muscular belly reaches 10% of the maximum displacement (10% Dm). Shorter Td indicate an earlier onset of contraction and a faster muscle reaction. Td is substantially related to the muscle fibre conduction velocity^36^, being both slower in slow-twitch than in fast-twitch fibres^40,41^. Fast fibres have been reported to store more elastic energy than slow fibers^42^, which enhances the speed of muscle relaxation and enables a faster realization of consecutive contractions^36^. As a consequence, athletes with shorter Td have been reported to show an increased ability to rapidly generate force during repeated muscle contractions^43^. Similarly, our result suggests that the futsal players with higher Td may have a lower ability for repeated high-intensity actions which is reflected by a higher decrement in RSA performance.

The results of the present study show the effects of a RSA test on the contractile properties and the neuromuscular profile of elite futsal players. Authors like De Paula Simola *et al*.^44^, or Wiewelhove *et al*.^21^ have already investigated the effects of fatigue on the contractile properties of the muscle in different sports. Although their results differ in many aspects, these authors generally agree that time-variables decreasing and the Dm increasing involve a normal response following the muscle training, especially in muscles adapted to explosive exercises^12^. The decrease in muscle stiffness evidenced by the increment of the Dm is produced as a consequence of fatigue and implies a loss of strength and explosive potential, reducing the ability to generate strength rapidly^45^. An unusual increase of the Dm can indicate chronic fatigue^35^, although this was not present in this study. Regarding the time variables (Td, Tc, Ts and Tr), the disparity between the described results of the previous research makes it difficult to draw conclusions, although most of the studies associated muscle fatigue with increased values of these variables. In the present study, the results show a reduction in all of the time parameters (Td, Tc, Ts and Tr) of the RF in both legs. This indicates that our RSA test did not induce muscle fatigue in the RF muscles of our participants but rather, it had a potentiating effect on their contractile properties^14,25^. The only symptom detected is the significant increase of the Dm in the RF of the dominant leg^46^. Regarding the BF, a significant reduction was observed in the Ts of both legs. The variability of this parameter and the possible influence of the co-activation of other neighbouring muscles during the TMG evaluation makes it difficult to interpret this behaviour^38^. Future studies with different protocols^47^ must be performed to characterise the performance decrement in futsal.

In the light of the results, we can conclude that the RSA test provokes an increase in the excitability of the RF in both legs (lower values for Td, Tc, Ts and Tr), while its effects on the contractile properties of the BF are less clear. Also, the RF of the dominant leg seems to be the first muscle to present performance decrement symptoms, as it is the first that starts to lose muscle tone (higher value of Dm) as a consequence of the effort. There was however, no effect on lateral symmetry. In line with the study by Gil *et al*.^23^ in Brazilian footballers, no significant differences were found either before or after the RSA test. Although the results are hardly comparable, there are very few studies that cover this topic in futsal players and the fact that no significant difference was found between the dominant and non-dominant leg could be considered as a health symptom of the sample, as an imbalance is associated with an increase in injury risk^20,48^. Further research is required to investigate the behaviour of these variables in real fatigue situations as this is when there is a higher risk of muscle injury.

There were moderate negative correlations (0.3–0.5) between the performance decrement (RSA_DEC_) and the variation of the Tc, as well as between performance decrement (RSA_CHANGE_) and the variation of the Dm in the the RF. These correlations indicate that a greater decrement (identified as a significant decrease in performance) will correspond to lower variations of the Tc and Dm in the RF. This means that the gap between the values measured before and after the RSA test decreases as performance decrement increases. Moreover, it would be expected that, in case of continuing the exercise until severe fatigue appears, post values will overcome pre values changing the sense of the differences, as described earlier by other authors^12,13,25^. Regarding the BF, the results of the analysis showed moderate-to-large positive correlations between the two performance decrement indicators (RSA_DEC_, RSA_CHANGE_) and the variation of the Td and the Tc, suggesting that the evolution of these variables (Td, Tc) may provide relevant information regarding the degree of fatigue in the BF muscles.

## Conclusion

The baseline contractile properties of lower-limb muscles are related to performance and the percent decrement in repeated high-intensity actions in futsal players. Lower Ts and Td baseline values in the BF were associated with a better performance and lower performance decrement in repeated sprint actions, respectively. Finally, greater changes in Td and Tc after the RSA test showed a relationship with a higher decrement in RSA test performance in futsal elite player. The behaviour of the contractile properties only describes the acute adaptations in the RF and the BF after completing the RSA test, as the measurement was made immediately after finishing the test. It will be necessary that future studies include neuromuscular parameter controls both 24 and 48 h after to understand the impact of fatigue on the contractile properties of these athletes across a longer time period.

Most studies about futsal have focused on analysing both the demands of futsal fixtures and the physiological profile of futsal players. This is the first research study to assess the effect of a repeated sprint test on the contractile muscle profile in elite futsal players. The findings suggest that muscle mechanical variables have a significant relationship with the performance during a RSA test. Therefore, tensiomyography is a useful instrument to assess the influence of contractile muscle ability on the physical performance and the performance decrement during in high-intensity actions of futsal players. The contractile profile of elite futsal players from Spain provided in this study provides knowledge about the muscle properties of healthy elite futsal players in the middle of the season. These results can be useful for physical trainers and coaches as a reference to design specific training and rehabilitation programs to reach the references values and an optimal muscle contractile ability. In short, this research supports the use of tensiomyography as a tool that is sensitive enough to detect mechanical changes and to aid in the understanding of how these changes affect the capacity of futsal players to perform intermittent efforts at a high intensity.
